# A pre-Hispanic canoe or *Wampo* burial in Northwestern Patagonia, Argentina

**DOI:** 10.1371/journal.pone.0272833

**Published:** 2022-08-24

**Authors:** Alberto E. Pérez, Rodrigo Moulian Tesmer, Juan Francisco Reyes Sánchez, José L. Lanata, Andrea Medina, Miguel Chapanoff Cerda

**Affiliations:** 1 Departamento de Antropología, Universidad Católica de Temuco, Temuco, Chile; 2 Instituto de Comunicación Social, Universidad Austral de Chile, Valdivia, Chile; 3 Equipo Chileno de Antropología Forense y Derechos Humanos (ECHAF), Bariloche, Argentina; 4 IIDIPCa-CONICET, Universidad de Río Negro, San Martin de los Andes, Argentina; 5 Cátedra de Dendrología, AUSMA-Universidad Nacional del Comahue, Neuquén, Argentina; 6 Museo Regional de la Araucanía, Servicio Nacional del Patrimonio Cultural, Temuco, Chile; New York State Museum, UNITED STATES

## Abstract

The burial of Individual 3 at the Newen Antug site, a young adult woman, with a pottery grave offering characteristic of the Late Pottery period and dated to 880 years BP, is an indirect burial in a wooden structure. The form and design comprise a wooden *wampo* or small canoe, or a symbolic representation of one, a metaphor in current and historical Mapuche society for the voyage to the final abode of the dead, located beyond a water body which must be crossed in a boat. This is the first find of a burial in a canoe structure in Argentinian Patagonia, and the most southern example on the whole continent. It is also the earliest record in Argentina of pottery of the Red on White Bichrome tradition used as a grave offering, extending the repertoire of characteristics shared between the two slopes of the Andes mountains during the pottery periods, including ritual as well as material aspects.

## Introduction

Canoes constructed out of a single trunk were widely used throughout the whole American continent [1]. In many societies whose way of life centred around water, either sea or lakes, canoe burials formed part of a set of funerary customs called “water burials” [2]. This author recognised that canoe burials in North America present different modes, which can be divided into: A- Aquatic: 1- placing bodies in boats and then setting them adrift on the water; 2- sinking the canoe in a water body; and B- Terrestrial: 1- placing bodies in canoes exposed to the elements (including the use of platforms, posts and branches; 2- in negative graves (excavations), i.e. below ground-level; 3- in positive graves (tumuli), placing the canoe on the ground and covering it to generate prominent superficial structures [2].

In the southern cone of the Americas, specifically in Northwestern Patagonia (Argentina) and La Araucanía (Chile), the historical, ethnographic and archaeological records mention all three modes of terrestrial burial. They describe the coffin-canoe being placed in trees or hung from posts, while some are covered with soil to create tumuli [3]. The earliest records suggest canoe burials in excavated graves in areas of social importance. Based on a reinterpretation of the archaeological contexts of the Padre Las Casas and Gorbea 3 funerary sites in south central Chile, some investigators have disputed the existence of canoe burials or the appropriateness of the term. They claim that the archaeological contexts of the region are ambiguous, since they mention only soil impressions, changes in soil colour and vestiges of wood which are insufficient to distinguish a coffin from a canoe. For example, none of these vestiges and structures present structural or design aspects suggesting that they were suitable for use on water, such as bow and stern [4]. According to Carabias et al. [5], scepticism was fed by the ethnographers Steward and Faron [6: 277]; these authors relativised the use of boats based on the scarcity of navigable rivers available to the majority of the indigenous population, who lived in inland valleys. Others postulate simply that dug-out (single-trunk) canoes were introduced into central Chile by the Spaniards [7: 54–58]. Lira [4] offers the alternative suggestion that these coffins were some kind of symbolic representation of nautical artefacts and navigation practices linked to funerary rituals. This hypothesis makes sense in view of the importance of water and navigation, given the shore-dwelling character of Mapuche society and its precursors [5, 8, 9]. In other words, water is a prominent feature of the natural surroundings, of sufficiently strategic importance for communities to adopt its forms and symbolisation [10] in a dendritic model of space and territoriality where aquatic landscapes have a profound meaning [10, 11]. Taking a different approach, Chapanoff [12] postulates that the evidence, be it archaeological or ethnographic and historical, is insufficient to sustain this idea.

**Fig 1:**
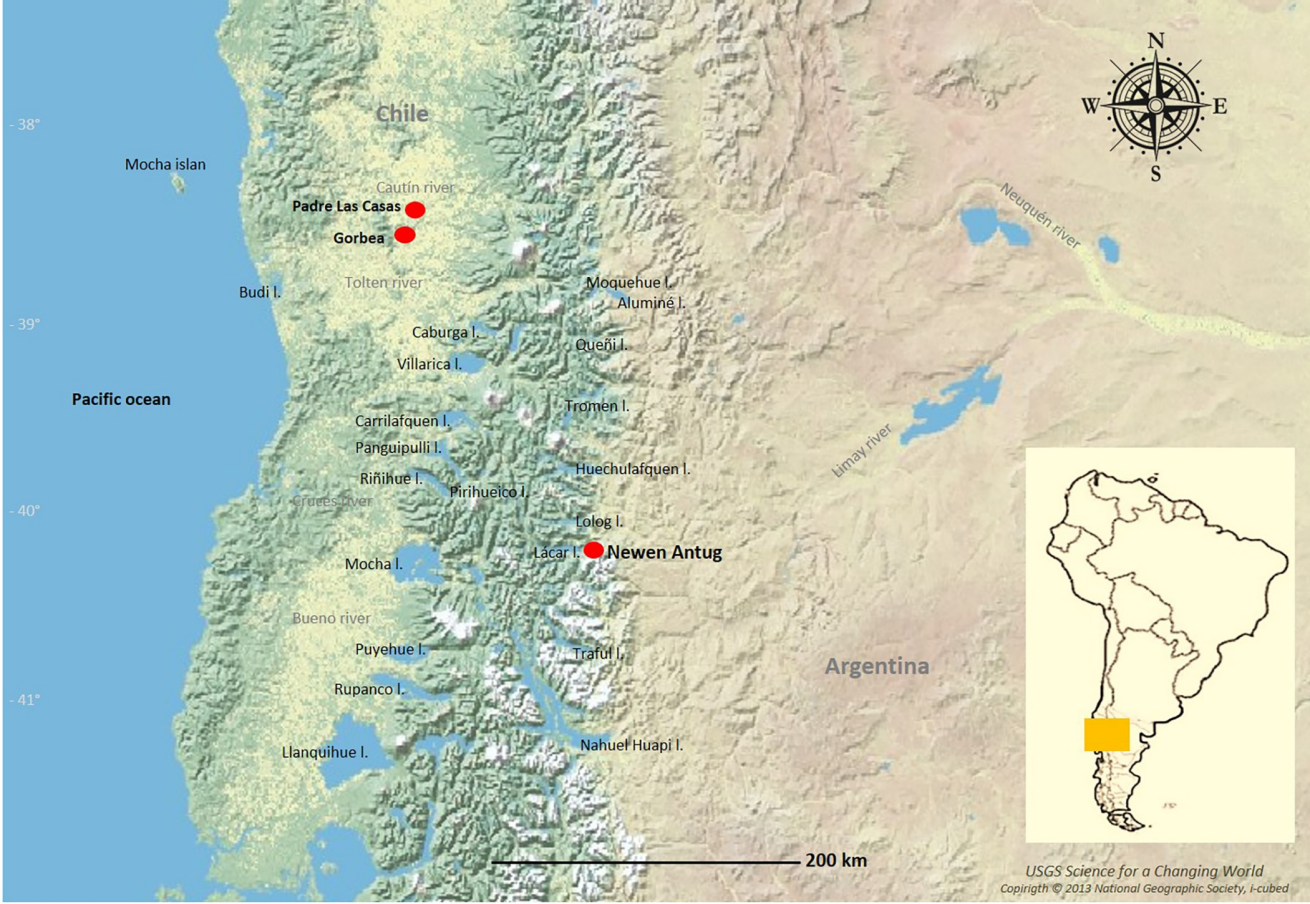
Main bodies of water mentioned and archaeological sites in red.

To clarify the real importance of navigation in mortuary rites, below we present the structure found in Burial #3 of the Newen Antug archaeological site, located in an area of Andean forests and lakes in the North Patagonian province of Neuquén, Republic of Argentina (Fig 1). Our hypothesis is that this is a simple, indirect burial, with the individual contained in a wooden structure. Given the position of the body and the artefactual, temporal and lakeside context with which it is associated, we postulate that this was a burial in which a canoe (*wampo* in Mapudungun, the Mapuche language) served as the coffin (*trolof*). This form of burial has great value and symbolic power in Mapuche society.

To test this hypothesis, we propose to discuss the navigating practices of this society and their antiquity from the archaeological record of south central Chile and Northwestern Patagonia in Argentina, part of the ancestral territoriality of the historical Mapuche people, defined by them as *Wallmapu*. We will go on to analyse a corpus of ethnohistorical and ethnographic information that provides evidence on material and immaterial aspects of current and historical Mapuche society, referring to navigation technology, burial practices, cosmovision and funerary rites. Our object is to try to characterise the importance of navigation technology in these societies; this may be represented in material and immaterial practices, for example by burying people in canoes, or representations of canoes, as a metaphor for the voyage to the final abode of the dead, located beyond a water body which must be crossed in a boat.

Considering the factors of equifinality that may exist in the identification of nautical artefacts like canoes and coffins constructed of hollowed-out tree-trunks (McGrail 1987 and Barnesley 2012, in [12]), we establish that one way of distinguishing or postulating them is through the context of these wooden artefacts and their association with aquatic media (material, symbolic and landscape/environmental).

### Newen Antug

**Fig 2:**
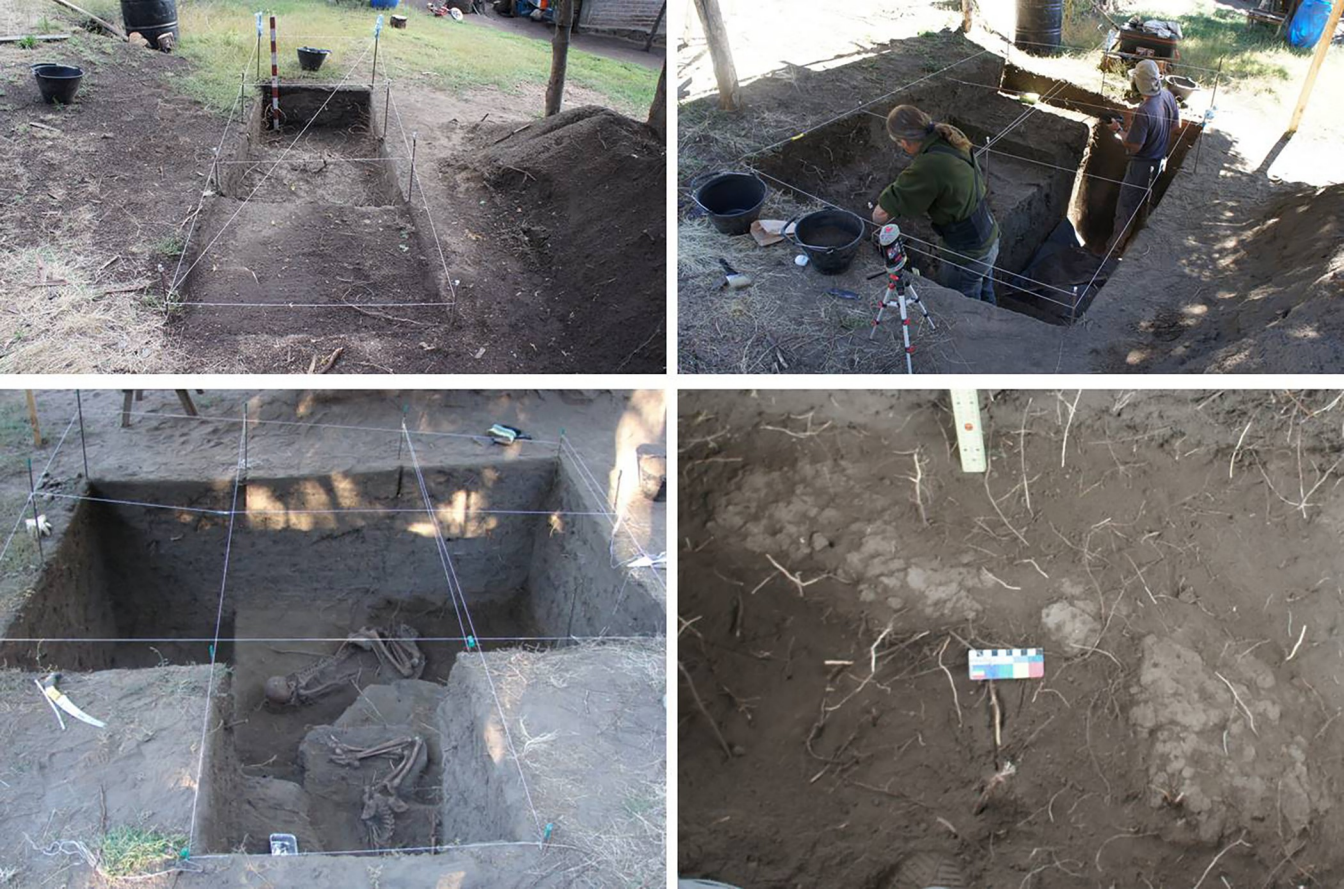
A, B Excavation of the Newen Antug site, C- human bodies, D- Stratum 5 of hardened soil and outline of inhumation pit.

**Fig 3:**
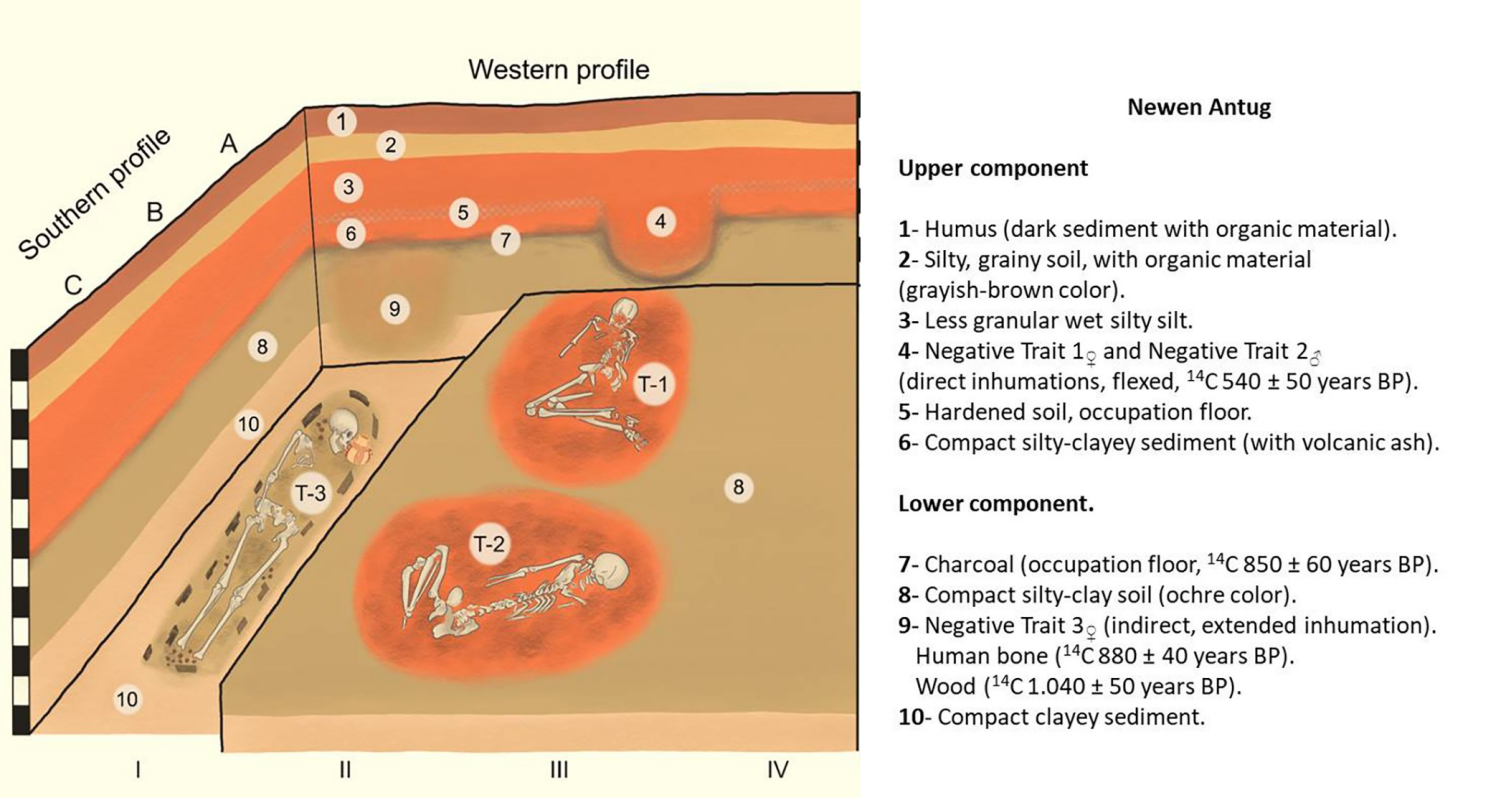
Excavation plan of the site.

Newen Antug (40° 09´ 44´´S, 71° 20´ 49´´W), is a multicomponent, open-air site located on Comandante Díaz mountain, on the south-eastern slope of the Chapelco Range, very close to Lácar Lake (Fig 1). The site is in an area of transitional mixed forest of Chilean cedar (*Austrocedrus chilensis*) and radal (*Lomatia hirsuta*). Ten 1 x 1 m squares were excavated in 10 cm-deep layers, allowing two cultural components to be identified (Figs 2 and 3). The soil matrix is fairly homogeneous, consisting of clayey sediments and volcanic ash, all fine-grade elements transported by wind and water associated with artefacts and geofacts (Fig 3).

The Upper Component was dated to 540 ± 50 years BP (LP 3024, charcoal) by ^14^C. It consists of a friable, porous matrix down to a depth of 80 cm below present ground level, presenting exclusively funerary characteristics due to the burial structures of Individuals 1_♀_ and 2_♂_. Both are buried directly in right lateral decubitus; one is a male individual associated with a pedunculate projectile point made of obsidian (proceeding from a source in Lácar Lake accessible by water) and the other a woman with a pottery grave offering and bone artefacts and ecofacts [13, 14].

The latest occupation date for the Lower Component is 850 ± 60 years BP (dated by ^14^C, LP 3271, charcoal); it extends from 0.80 m to 1.30 m depth and its composition is a continuation of the top matrix, but damper and more compact [13, 14]. This component includes residential and funerary occupations associated with the Late Pottery period [15, 16].

In the present work we present a description of the structure of Burial #3, Individual 3_♀_, which forms part of this occupation, although according to the ^14^C results it is older: 880 ± 40 years BP (LP 3426, human bone, Individual 3_♀_) and 1,040 ± 50 years BP (LP 3411, wooden artefact associated with the human body). This individual is lying stretched out straight, her cranium and forearms are decorated with red colorant, and she has a pottery grave offering of the Red on White Bichrome tradition. Inside the grave, the body was lying on top of valves of freshwater molluscs and surrounded by pieces of wood. The excavation of individual #3 of the Newen Antug site was part of a rescue work carried out by technical staff of the Archaeology and Ethnohistory Laboratory of the Secretariat of Planning and Sustainable Development of the Municipality of San Martin de los Andes, Province of Neuquén, with the consent of the Mapuche Curruhuinca Community. The materials from the site, including individual #3 and its associated artifactual context are in the premises of the Municipality of San Martin de los Andes, Province of Neuquén, Argentina.

#### Burial #3, Newen Antug

The body was disposed whole-body in the east-west profile between grids A and B, at a depth of 1.30 m. The individual was sexed on available segments, skull base and pelvic morphology [17]. Similarly, age was estimated from cranial and postcranial skeletal metamorphosis and dental wear [17]. Height was estimated from skeletal measurements (heel to vertex) in situ. Individual 3_♀_ is a juvenile or young adult, 17 to 25 years old, female, and measures approximately 149 cm. There are traces of red dye on the skull and forearms. Among the items found that accompanied or contained the body are a ceramic vessel, 11 freshwater mollusk shells, and over 500 fragments of plant material.

#### Plant material

At depths between 1.30 and 0.98 m, exclusively restricted to locations beneath (23%) and forming a perimeter lateral to the body (77%), at least 578 fragments of plant matter (wood) were recorded, taking a macroscopic form of black and brown cubes. The sizes of the fragments were classified into Small, between 1 and 3 cm (341 fragments), Medium-Small 3 to 6 cm (89 fragments), Medium, 6 to 10 cm (61 fragments), Large, 10 to 12 cm (34 fragments), and Very Large, greater than 12 cm (53 fragments), the latter concentrated in distal sectors.

**Fig 4:**
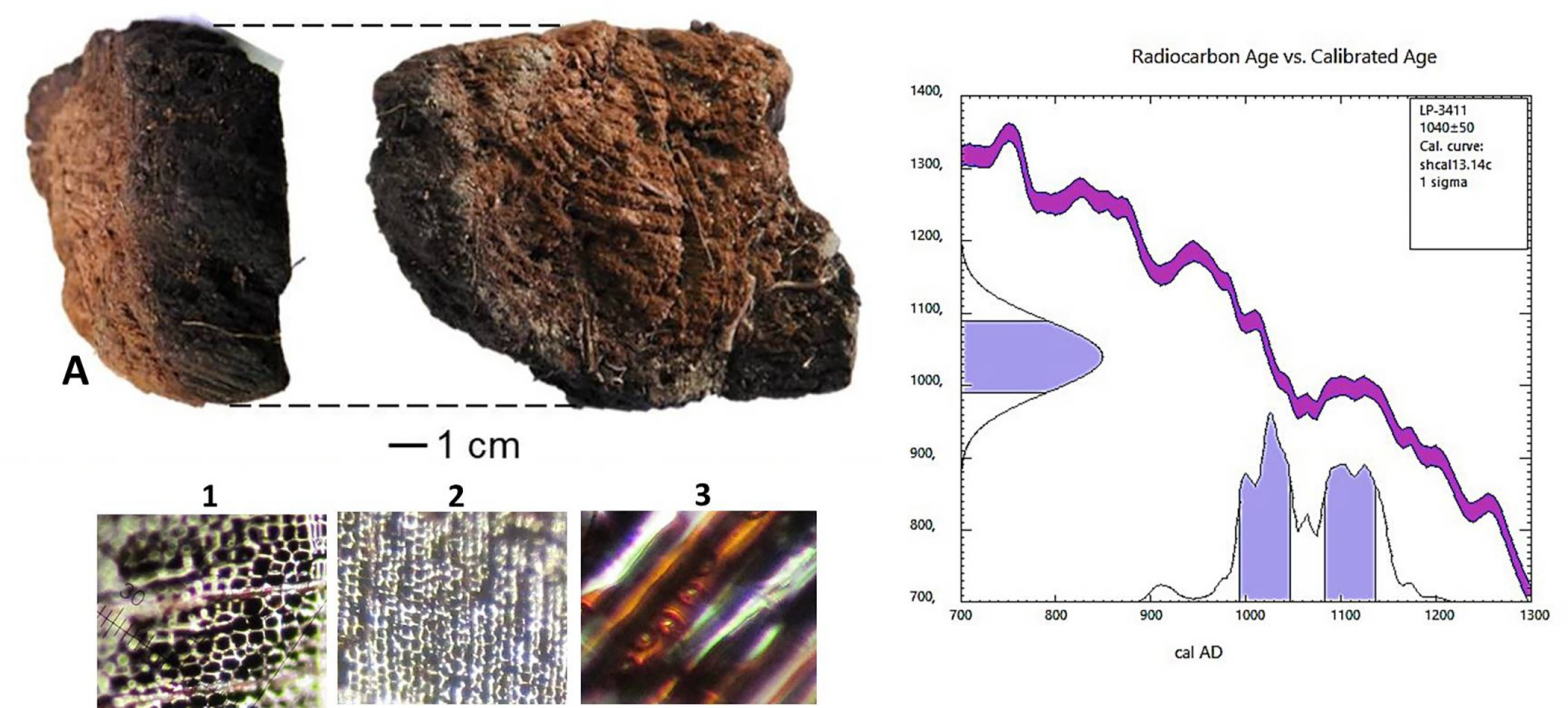
A- wood with charred face and ^14^C (S1 Fig) dating. Sections: 1- cross, 2- radial axial, 3- tangential.

A sample of 20 fragments of wood of different sizes and from different sectors was selected and sent for identification in the Laboratorio de Dendroescrerología y Cátedra de Anatomía Vegetal del AUSMA, Universidad Nacional de Comahue, Argentina. The wood was observed under an optical microscope with incident light in the three diagnostic sections: cross (Fig 4A; S2 Fig), radial longitudinal or axial (Fig 4B; S3 Fig) and tangential longitudinal or axial (Fig 4C; S4 Fig). It was concluded from the observations that the wood came from a Gymnosperm (clearly tracheid wood). In the radial section, round or oval tracheids with uniseriate pits were observed (Fig 4B), indicating that the wood is from *Austrocedrus chilensis* “Chilean cedar”. Among the characteristics of the sample, it was noted that 31% of the Large and Very Large specimens, i.e. those big enough for superficial modifications to be observed, present heat alteration exclusively on the face oriented towards the body, or internal face in the case of a container. Despite the fragmentation of the sample, the structural characteristics of the fragments suggest that they are drawn from a single tree, and projection of the angles of the growth rings indicates a single trunk of diameter larger than 0.70 m (Fig 4A).

#### Malacological elements

The body is accompanied by valves of the freshwater mollusc *Diplodon chilensis*, some of which are semi-complete and lie below and in direct contact with bones such as the humerus and femur. The shells conserve a degree of integrity and fragments of periostracum are present, from which it is inferred that they were placed on the base of the wooden container before the body was laid in it. Three semi-complete specimens present red mineral colorants on their outer surface. This species of mollusc is found only 300 m away on the shores of Lácar Lake. Valves of this species are recorded in funerary and residential contexts in sites on both slopes of the Andes [18, 19].

#### Pottery

**Fig 5:**
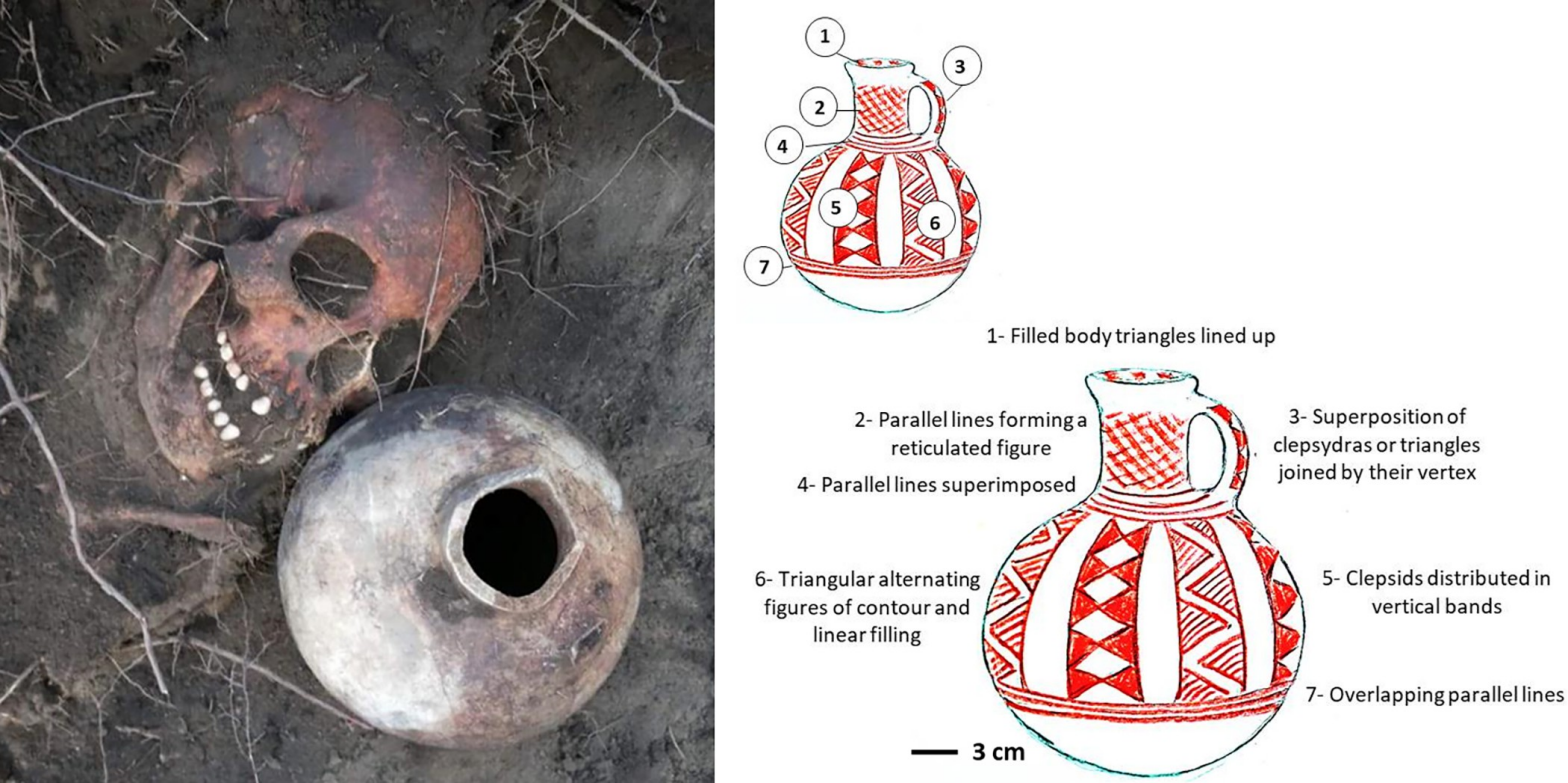
El Vergel pot from Burial N° 3.

Beside the body, to the left of the cranium and on the same vertical level, a pottery vessel was deposited [20]; it is a bottle or jug with a spherical body and a formatted or flat base, cylindrical neck, and mouth with signs of a pouring-lip. It has a handle consisting of a vertical strip fixed between the body and the rim, opposite the lip. It was fired with oxidation; the clay is compact and schist-like, red in colour with quartz inclusions (Fig 5). It has a white glaze and geometric patterns in red paint on several distinct panels on the body and neck. The motifs are: parallel lines dividing the body and neck into horizontal segments; reticulated figures round the neck; and vertical parallel bands distributed round the body where triangular figures composed of lines alternate with clepsydras or triangles joined by their vertex. This piece forms a typological or singular group (bottle or jug with narrow mouth and pouring-lip), previously unknown at least among the morphological and technical variants of the El Vergel pottery complex [21]; it is part of the (pre-Hispanic) Red on White Bichrome tradition, of the Late Pottery period in the regional ceramics sequence [22]. Given the chronology of this piece, it is one of the earliest records of use as a funerary item. Like other features, its tilted position and height, close to the face of Individual 3_♀,_ suggest that the wooden container was concave in shape and of restricted size, and that the base in this part was elevated.

#### Position of the body

**Fig 6:**
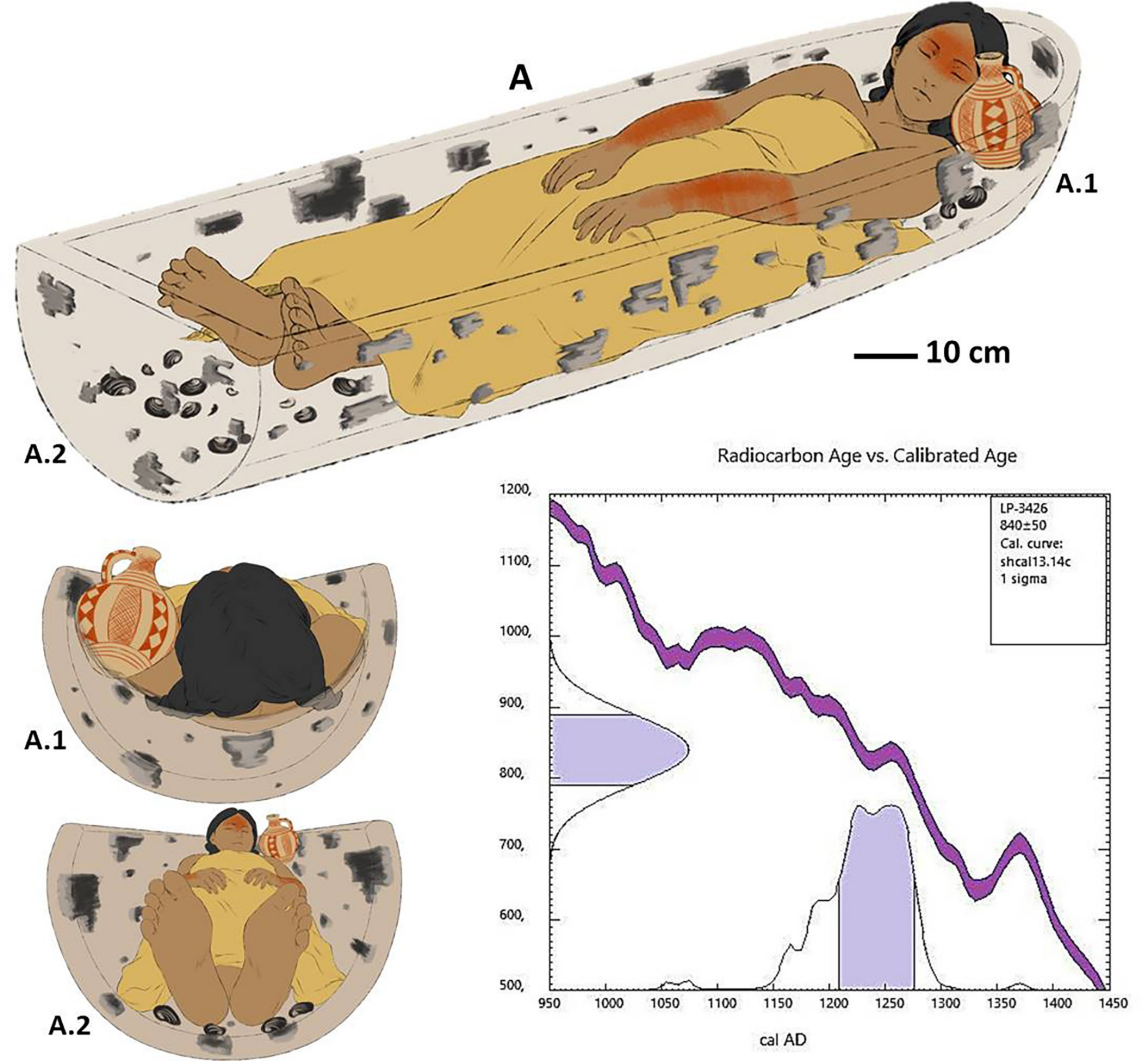
Reconstruction of the position of the body, associated artefacts and ^14^C dating of the body (S5 Fig).

The skeleton was found in dorsal decubitus, with extended legs, arms parallel to the axis of the body and hands in the pubic area in pronation. There is slight hunching of the shoulders (without rotation) and compaction of the thorax to medial (greater destruction and disorder *in situ*). The cervical portion of the spine was found to be flexed, showing that the chin (mandible) was inclined towards the sternum; the thoracic and dorsal parts of the spine lay flat on the floor of the grave, while the cranium was 26 cm above it. There must therefore have been some kind of structure on which the head and neck (and the pottery vessel) rested, of which only degraded vestiges of wood remain (Fig 6).

**Fig 7:**
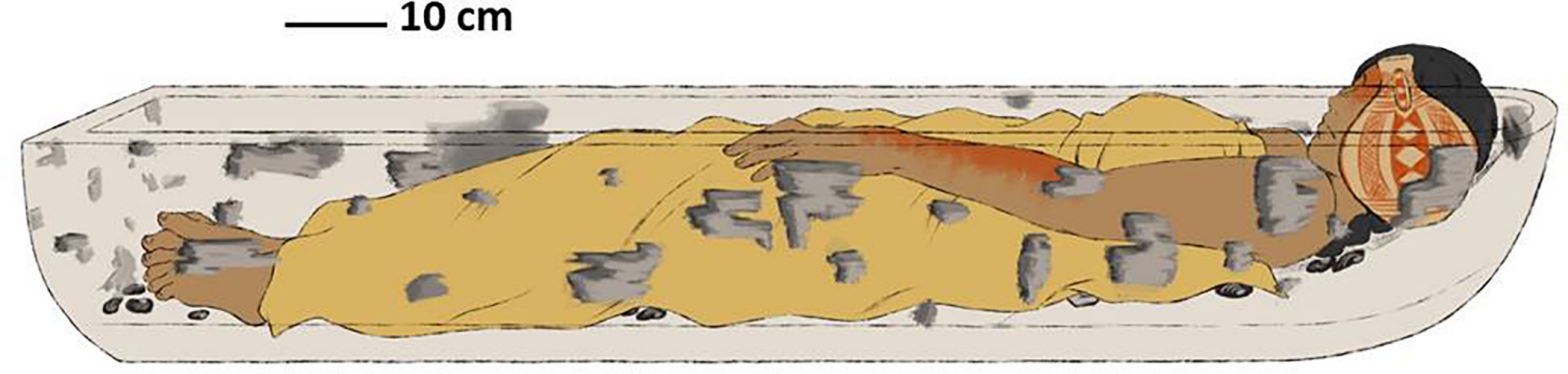
Recreation of lateral view in which bow and stern can be distinguished.

The body is laid out straight on the deepest level of the grave (1.40 m below current ground level), with a slope of 7 cm between the distal sector (1.33 m) and the proximal (1.40 m); its total length is 1.49 m. The gradient in most of the body is -3°, but between the shoulder blades and the occiput it increases to 25°, raising the shoulder blades, clavicles, cervical vertebrae and cranium above the rest of the body by up to 26 cm. The cranium was found between 1.18 and 1.27 m below ground level (Fig 7).

### Records of canoe or *wampo* burials

The use of funerary containers of worked wood, typified as ‘canoes’ (Mapudungun *wampo)*, is well established in the archaeological repertoire of the Mapuche culture [23–27]. This method of burial dates from 1280 ± 80 AD [26]. In the 19th and early 20th century it was the predominant procedure for indirect burial [28–33], including records from the east slope of the Andes [34]. References persist in the oral memory of ethnographic communities [6, 35–37].

The *wampo*, also called *trolof* in Mapudungun, is made from a tree-trunk, generally *roble* or *pellín* (*Nothofagus obliqua*), split into two and hollowed out two make two sections of coffin. One contained the corpse and the other was used as a lid. According to Erize [38: 100] these were called respectively *chraoguenel* and *chraiguenel*. Nevertheless, in some cases the coffin consisted only of the former [31] or only of the latter [39]. While the archaeological record shows a greater variability of species used, including coihue (*Nothofagus dombeyi*), raulí (*Nothofagus alpina*), ulmo (*Eucyphia cordifolia*) and laurel (*Laurelia sempervirens*) [4].

**Fig 8:**
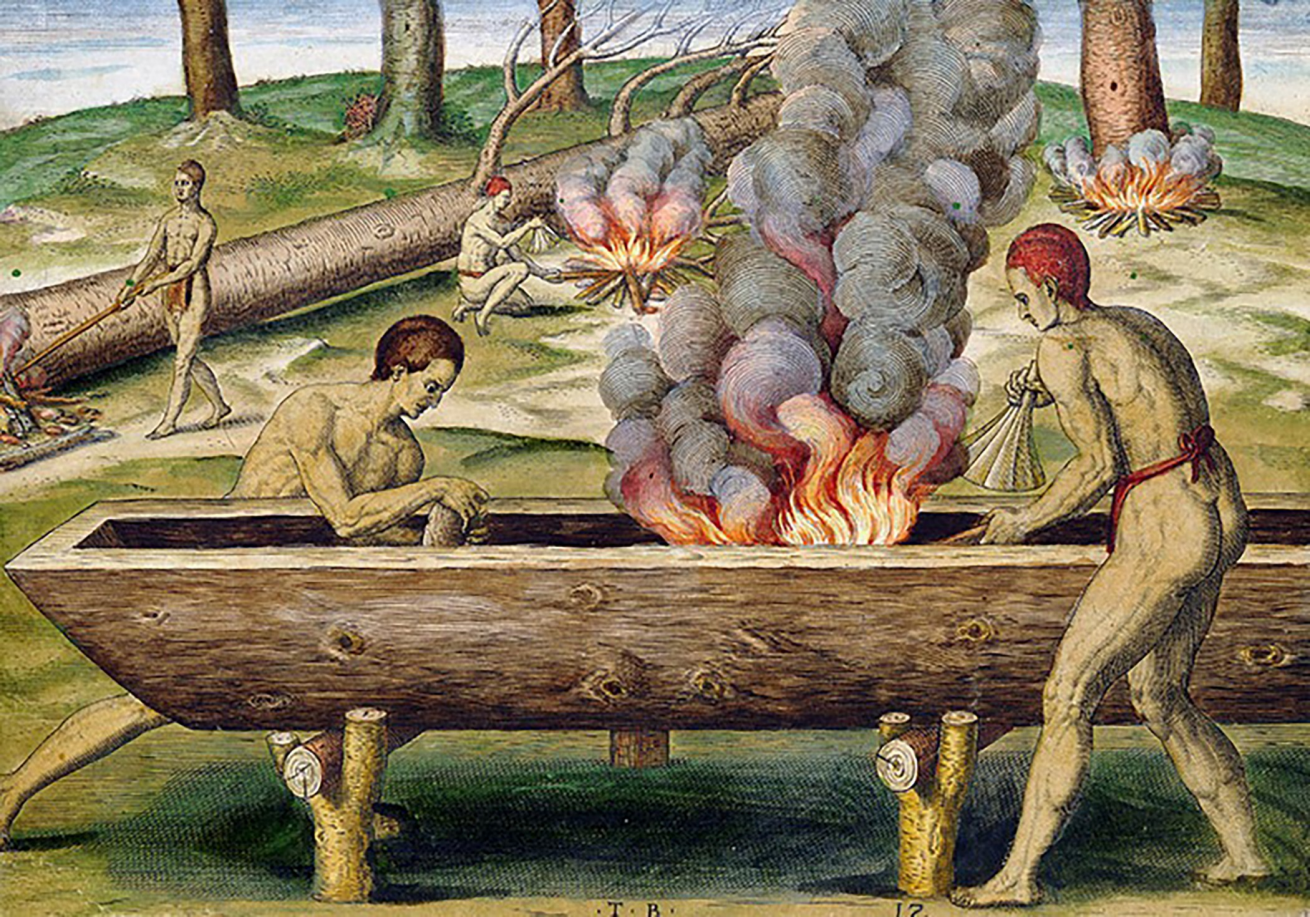
Mapuche burial, c. 1900 (in Chapanoff 2020: 14). The photograph shows a funeral ritual with a *wampo* beside the *rewe*.

In the early 20th century, Robles [39] reported that at the burial of *Cacique* (chief) Huilío Lienan in the *eltun* or cemetery in 1904, “the *wampo* was waiting beside the newly-dug grave” [39: 179] and was used to cover the corpse and the grave offerings. In the same report, Robles [39: 182] states that the *eltun* was clearly marked by the presence of “Latin” or “Greek” crosses, and “crude [anthropomorphic] figures of carved wood” and funerary canoes “that they place on the grave like a gravestone” (Fig 8).

The term *wampo* or *huampo* is documented from an early date by ethnolinguistic sources. Valdivia [40] records this word with the meaning of ‘boat’ and ‘ship’, and the same is found in Febres [41], Havestadt [42] and Augusta [43]. It is in fact a Quechua word incorporated into Mapudungun [44–46]. The linguistic repertoire reported by Valdivia [40] also includes the word *huampelleun* to mean ‘watch over [a corpse]’. The reconstructed form for analysis of this term is *wampülluwun* (*wampo-püllu-wun*), which means literally ‘remaining until dawn with the spirit in the canoe’. This is consistent with the Mapuche custom of spending all night watching over their dead. The inclusion of the word *wampo* in the funerary linguistic repertoire remains current in the Mapuche culture, where it means ‘coffin’ [Coña in 33, 47, 48]. Furthermore, *wampongün* means to ‘place the body in the coffin’ [47: 203].

Latcham [31] remarks on differences between the designs of Mapuche boats and the coffins called *wampo*, as the latter usually have flat ends. This distinction has been emphasised by Lira [4], Carabias, Lira and Adán [5] and Chapanoff [12]. Nevertheless, contextual analysis of Mapuche eschatological representations [see 49] indicate that the designation of the coffin by the term *wampo* has a metaphorical connotation. One of the conceptions of the passage to the next life in this society includes the voyage down rivers and across the sea to reach the final abode.

*Nomelafken*, the other side of the sea, is identified as a destination of souls [30, 32, 39, 50–52]. The spirit of the dead travels across the water to an island called *Külchemapu* or *Külchemaiwe*. Sanfuentes [53: 143] reports: “The Araucans site the graves of their dead on the bank of a stream to allow the current to carry the soul to the land of souls, which some believe to be Mocha Island”. The term *amunkowe*, the ‘place where the waters go’, expresses this idea symbolically. For this reason, defence of the free course of rivers is a cultural claim raised in today’s environmental conflicts.

In the framework of the Mapuche cosmovision, the *wampo* or canoe is conceived to be a means for passing to the other life [8, 32, 35, 47, 48, 54]. The mythical figure of the ferryman (*nontuefe*) is a porter who helps souls through this ordeal [55]. His services are paid for with *llangka*, blue-coloured stones considered of great value. This transitional voyage feeds Mapuche oral narratives [56], which describe souls travelling in *wampos* down subterranean rivers. The same means are used by the dead to return to this world, as is described in the folk-tale of the *kaleuche* [57], known as the ‘ghost-ship’. The literal meaning of the word in Mapudungun is ‘people of another river’, alluding to the space between the corporeal and the spiritual life, whose borders are crossed in a boat.

This cultural metaphor of the *wampo*-coffin has remained fixed in popular Chilean culture in the expression ‘*creerse la muerte en bote*’ [‘thinking he is the dead man in the boat’], to talk about a presumptuous individual. This image refers to the Mapuche custom of dressing the dead in their finest clothes and putting their most treasured possessions in their coffins when they set off on their voyage to the other side. This practice manifests two features of Mapuche eschatological representations: on the one hand it shows death conceived of as a sort of continuation of life, in which the soul needs the material elements that it possessed on earth; on the other it highlights the processual nature of death, understood as a transition to the ‘destination of souls’ over a distance that must be crossed using the *wampo*.

### Navigation technology and boat use

The evidence collected can be divided into indirect and direct. In the former case, the existence of navigation technology can be inferred indirectly on the east slope of the Andes from island occupations dated to 2000 years BP in Nahuel Huapi Lake, Argentina [58, 59], and from portage sites evidenced by paths cleared of rocks below the current water level [60]. On the west slope, there is evidence that Mocha Island, 30 km off the Pacific coast of Chile, has been inhabited for more than 3,300 years [61]. Although no chronology has been defined, the direct evidence of the use of navigation technology consists of more than 30 dug-out canoes sunk in freshwater bodies on both slopes of the Andes [4, 59].

#### Boat types

Based on ethnohistorical and ethnographic evidence, Lothrop [62: 233–235] distinguishes two types of dug-out boats of pre-Hispanic origin in south central and southern Chile: "dug-out boats with pointed bows and square sterns" and "dug-out canoes with both ends pointed"; the former type is most common in south central Chile. In historical times, however, the documentary sources mention the presence of at least three types of boats for Patagonia and La Araucanía (Pacific Ocean and inland waters of the Andes mountains): 1- the *dalca* (of stitched planks), 2- the bark canoe and 3- the dug-out (single-trunk) canoe [59]. Historical sources refer mainly to the first two types, while evidence of the third is mainly archaeological [4, 59, 63]. Various types of nautical artefacts are described for La Araucanía in Chile and the Lácar Basin (Argentina) in the eastern part of the Valdivian Basin. The most important are dug-out canoes of various sizes, including designs associated with different functions and aquatic environments.

### The ethnohistorical record of nautical activity

In the western part of the Valdivian Basin, in Chile, navigation in canoes was described in the 16th century on Villarica Lake [64: 157] and further south on Ranco [65: 402] and Llanquihue Lakes [66: 30]. In the first two cases there are also records originally dating from the 17th century [50, Vol. I: 176 and 255]. The same author mentions the use of *dalcas* or *piraguas* in mountain water-bodies close to Chiloé [50, Vol. I: 176]. In 1877, there is a report of indigenous canoes hewn roughly from a single trunk in both Bueno and Ranco Lakes [67: 36].

According to Carabias et al. [5], dug-out canoes were used in south central Chile for: 1- crossing or fording rivers and lakes; 2- transporting people and produce [66: 50, 6: 139]; 3- fishing, hunting and gathering [50, Vol. I: 174; 68: 141]; 4- strategic-military purposes and exploration [66: 30; 64: 158], including naval confrontations on Budi [64: 185–188] and Ranco Lakes [50, Vol. III: 394–397].

An important quantity and variety of information has been obtained in the pre-cordillera area of Calafquén, Villarrica and Caburga Lakes regarding the manufacture and use of canoes, *wampos* or *canogas*, as they are called by informants and sources [5]. It includes wood selection and manufacturing tools and techniques, as well as routes followed and the location of old ports [5].

We have ethnohistorical information on how the dug-out canoes of south central Chile were constructed from the Jesuit Diego de Rosales dating to 1678:

*The other boat much used in this kingdom is the canoe*: *they fell a tall*, *thick tree*, *cut away the side of the trunk that will serve as the keel or floor*, *hollow out the heart to leave the floor four fingers thick and the sides a little more than two*, *and shape the hollow trunk to serve as a boat*, *the narrower end for the bow and the thicker end for the stern* […] (Rosales 1877, Vol. I: 173–174)

On the east slope of the Andes, Rosales describes the use of *dalcas* or *piraguas* on Nahuel Huapi Lake, now in the province of Río Negro, in the 17th century (51, Vol. I: 176); and also naval confrontations involving dozens of dug-out canoes on Huechulafquén Lake [50, Vol. III: 394–397]. Various archaeological boats [59, 69] and portage sites [60] have been located in different parts of Nahuel Huapi Lake. Between 1861 and 1862, the Chilean expeditionist Guillermo Cox described the presence of dug-out canoes on Lácar Lake [70:148]. When he travelled up Pirihueico Lake, a few kilometres further west on the Chilean side of the Andes watershed, he observed the construction of a canoe but gave no details; however, in another part of his diary he adds that ‘the poor people’ construct canoes of *coihue* (*Nothofagus dombeyii*), hollowing out the trunks with fire and very simple tools [70:64]. In the Lácar basin we find the place-name *Nonthue*, which means ’ferry-crossing’; it is described by Larminat [71], who also photographed and illustrated a dug-out canoe [59, 71]. Finally, in the 1950s Ines Hilger [34] recorded the presence of burials in “*wampu*” in the Quila Quina peninsula on the south-east coast of Lácar Lake.

#### Variations in burial patterns in south central Chile

The archaeological record of south central Chile presents some of the oldest occupations and sites in the Americas [72], like Monte Verde I dated to 14,800 years BP [73]; however, the presence of human remains from which burial forms or patterns can be inferred go back to 9,500 years BP, presenting almost synchronic shared characteristics between coastal and mountain sites [74]. Throughout a large part of the Holocene, including the first millennium AD, burials in Pottery period contexts were direct, i.e. the bodies were placed directly in the earth. During the Late Pottery period, however, the start of which is at present considered to be related with the presence of Red on White Bichrome pottery from the 11^th^ century AD, indirect burials appear [14–16]. They take various forms, which may be summarised as (a) burials in a cist or rock-lined cavity [3, 32, 75, 76]; (b) wooden canoes or coffins made from hollowed-out tree-trunks [3, 23, 26, 25] and (c) burials in pottery urns [76–78].

## Discussion

Dendrological analysis enabled us to determine that the vestiges of wood were from the species *Austrocedrus chilensis* or Chilean cedar, a tree available locally with excellent buoyancy, known to have been used in navigation technology.

Unlike other contexts at the same site (Individuals 1♀ and 2♂), Individual 3♀ was indirectly buried, i.e., it was not deposited on the ground itself, but lying in a long, wooden containment structure (hollowed tree trunk) with a restricted, concave interior space. As a result, the skeleton was preserved with the bones extended and articulated, the shoulders hunched and the arms and forearms placed over the torso and hips.

The body follows the 3° inclination of the grave floor, with a sudden upward turn at the level of the shoulder-blades; these are opened, raising the clavicles, cervical vertebrae and cranium above the rest of the body at an angle of about 25°. Since several of these elements are in direct–albeit discontinuous–contact with wood, it would appear that the body rested on this containing structure which was tilted towards one end, suggesting that the head is at the bow and the feet at the stern.

**Fig 9:**
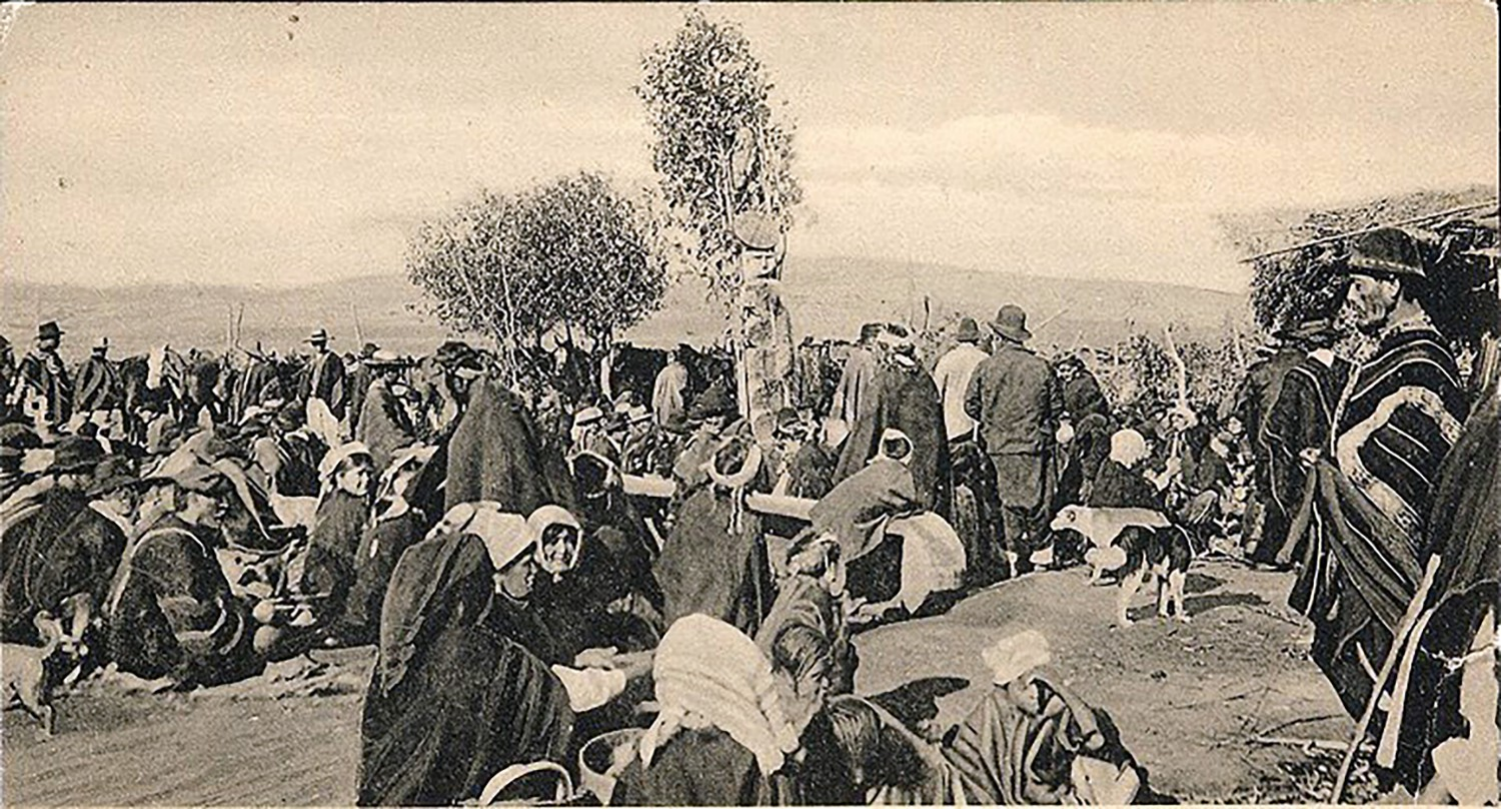
Construction of dug-out canoes in the chronicles of Virginia. Printed hand-coloured engraving by Theodor de Bry after John White, ca. 1590.

Analysis of the wood indicates that it is a single trunk which contained and supported, but did not cover, the extended body of Individual 3_♀_. The wood fragments are less thick in the lateral sections and thicker at the distal and proximal ends of the body, i.e. the bow and stern of the boat as described in the chronicles [50, 62]. Another characteristic is the heat alteration (charring) restricted to the inner surface of the wood; this would support the interpretation of a single trunk hollowed-out by the use of fire, just as 17th and 19th century chronicles mention [50, 70, 79]. Robles [39] observed the canoe burial of a woman in Budi Lake; the name given to the structure was *lufco* (*lüf*: “burning”, *co*: “water”), perhaps indicating a Mapudungun word for this burning technique (Fig 9).

## Final considerations

Individual 3_♀_ buried at the Newen Antug site is a young adult woman; her cranium and forearms are decorated with red colorant, and she has a pottery grave offering characteristic of the Late Pottery period. The dating of this piece to 880 years BP makes this the earliest record in Argentina of the use of Red on White Bichrome tradition pottery, and of its use as a funerary element. The burial is indirect, since the body and offerings lie within a wooden structure hollowed-out and shaped by fire.

This find extends the repertoire of characteristics shared between the east and west slopes of the Andes during the Pottery period, including both ritual and material aspects. Analysis and comparison of eyewitness reports and ethnohistorical and ethnographical information of current and historical Mapuche society show that the navigation technologies, burial practices and funeral rites employed here form a cosmogonic corpus which pre-Hispanic archaeological data indicate to be of great chronological and cultural depth. This whole corpus of information is indirect evidence of the existence of navigation technologies in the area contemporary with the burial of Individual 3_♀_, since it displays material and immaterial aspects of the eschatological representation of an artefact used as a coffin. In other words, the form and design of the coffin are those of a *wampo* or small canoe, or a metaphorical representation of one. Burial in a *wampo*, as given to Individual 3_♀_, is a metaphor of the dead soul’s transition or voyage down rivers and across the sea to its final abode. The Mapuche people recognise the limitations of the dead person on this water-borne voyage, and the resulting need for ornamentation, provisions and a boat.

To sum up, we present here the first find of a burial in a canoe structure, or its symbolic representation, in Argentinian Patagonia, and the most southern example found to date on the whole continent.

## Supporting information

S1 Fig LP-3411, Radiocarbon age and calibrated age of wood container from Individual 3_♀_. (JPG)

S2 Fig Full tangential cross section view of wood. (JPG)

S3 Fig Full cross sectional view of wood 1. (JPG)

S4 Fig Full cross sectional view of wood 2. (JPG)

S5 Fig LP-3426, Radiocarbon age and calibrated age on Individual 3_♀_ bone. (JPG)
