# Supplementary figures and images for: A pre-Hispanic canoe or Wampo burial in Northwestern Patagonia, Argentina
Source: PLoS One. 2022 Aug 24;17(8):e0272833. doi: 10.1371/journal.pone.0272833 (PMC9401108; doi:10.1371/journal.pone.0272833)

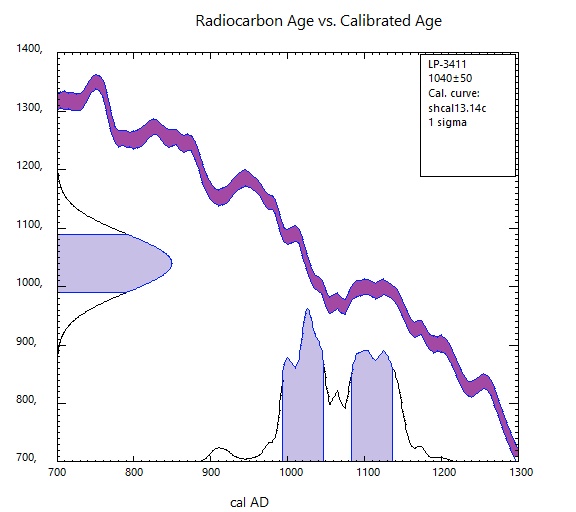

Supplement: S1 Fig — (JPG) [file pone.0272833.s001.jpg]

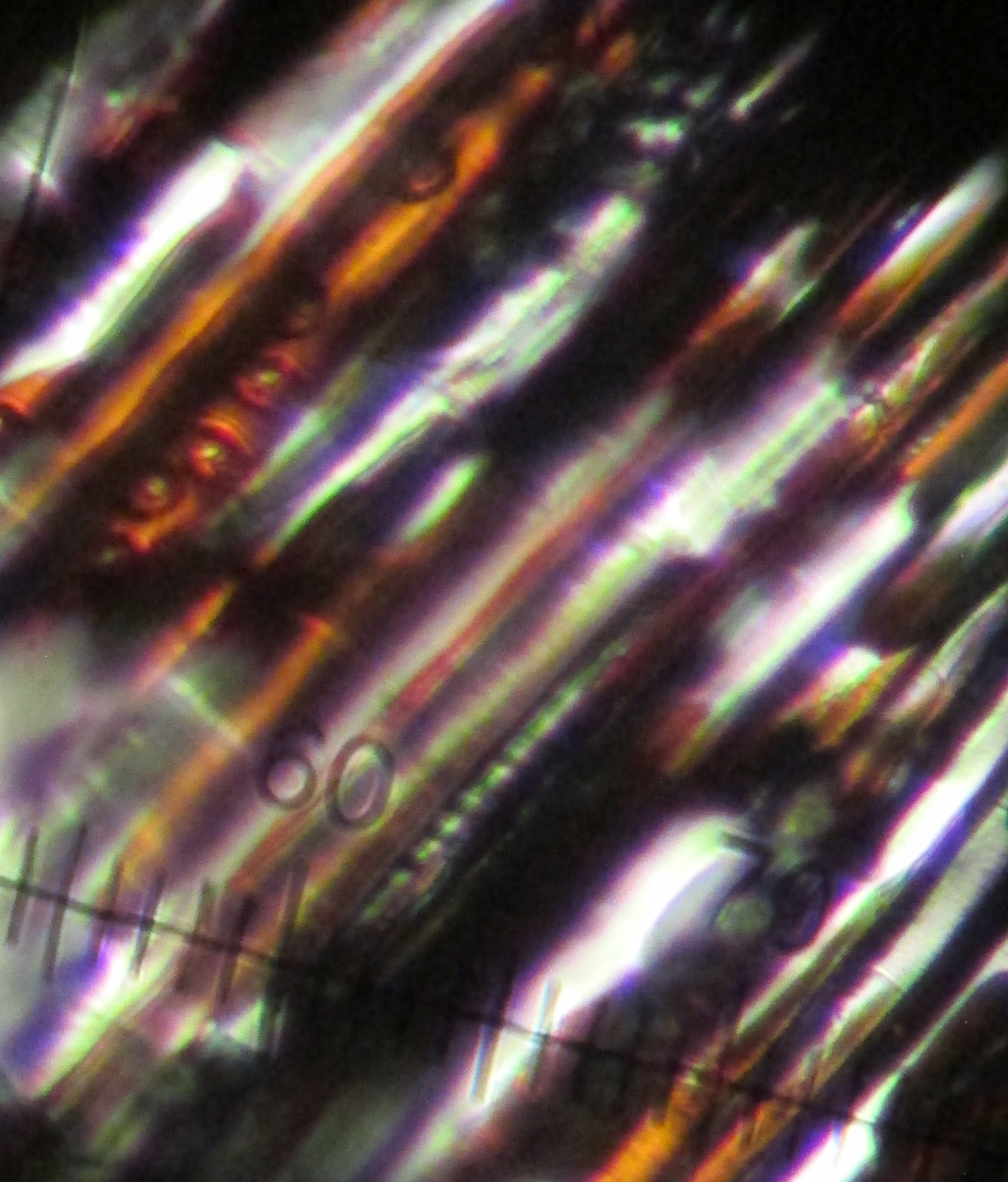

Supplement: S2 Fig — (JPG) [file pone.0272833.s002.jpg]

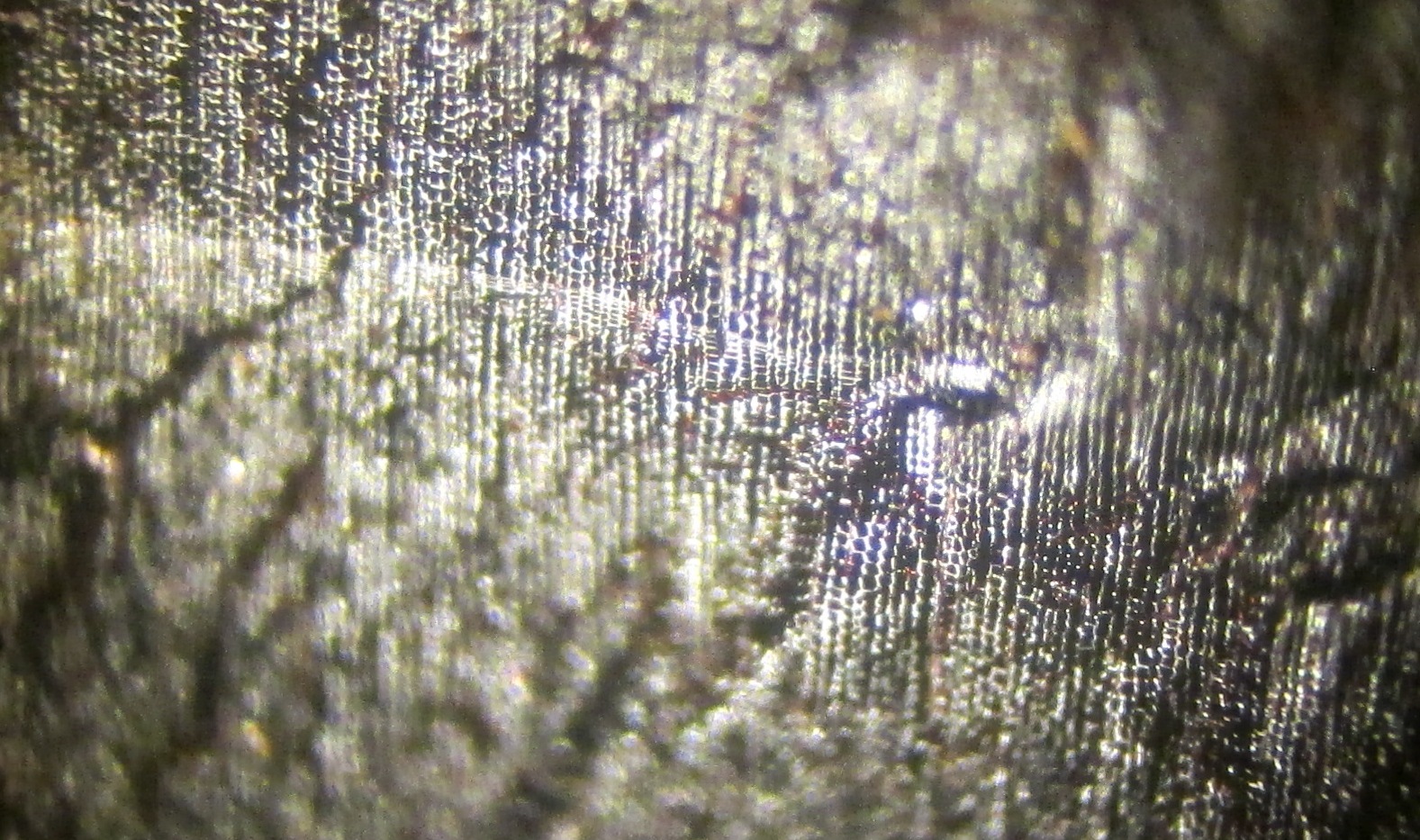

Supplement: S3 Fig — (JPG) [file pone.0272833.s003.jpg]

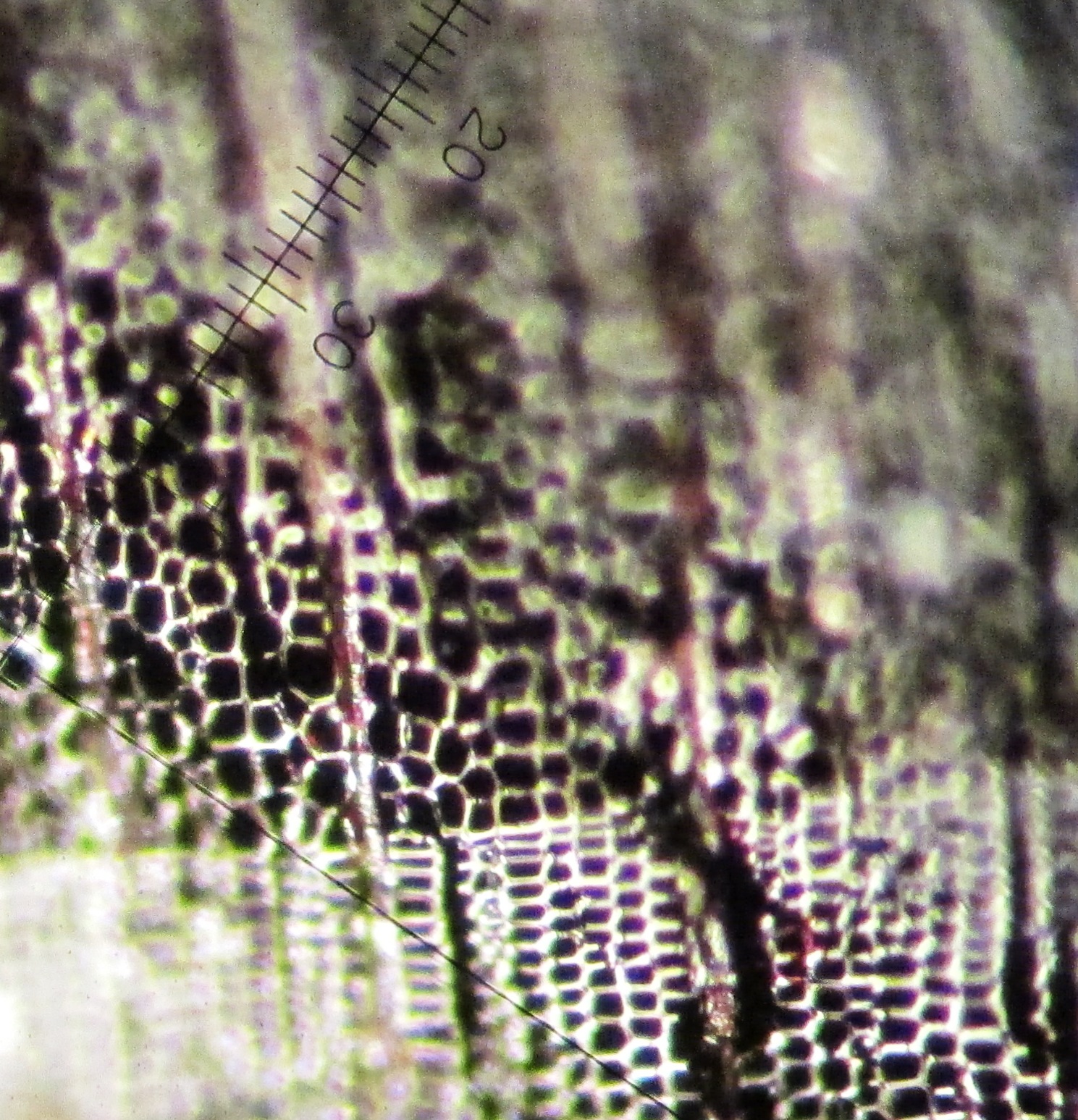

Supplement: S4 Fig — (JPG) [file pone.0272833.s004.jpg]

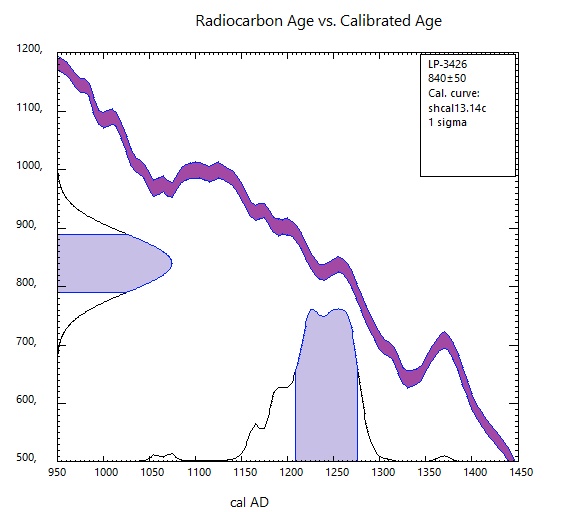

Supplement: S5 Fig — (JPG) [file pone.0272833.s005.jpg]
